# Gingival Cyst of the Adult: Report of an Inconspicuous Lesion Associated with Multiple Agenesis

**DOI:** 10.1155/2017/4346130

**Published:** 2017-03-09

**Authors:** Juliana Mançano Melhado Brod, Fabrício Passador-Santos, Andresa Borges Soares, Marcelo Sperandio

**Affiliations:** São Leopoldo Mandic Dental Institute and Research Center, Campinas, SP, Brazil

## Abstract

Gingival cyst of the adult is a rare slow growing and asymptomatic lesion that arises from the rests of the dental lamina. The present report describes the case of a miniscule adult gingival cyst in the lower anterior gingiva in a 51-year-old male with agenesis of lower premolars and lateral incisors. This paper contrasts the literature concerning the differentiation between the gingival cyst of the adult and the lateral periodontal cysts as well as the possible misguided concept that the former may be such rare an occurrence.

## 1. Introduction

Gingival cyst of adult is a rare odontogenic cyst from the rests of dental lamina [1]. It usually occurs in the soft tissues in the canine and premolar regions of the mandible and less frequently in the maxilla. Clinically, the lesion is often less than 6 mm in diameter and appears as a painless small flesh colored swelling, sometimes with a bluish hue due to the cystic fluid [2]. In some instances, the cyst may cause a scoop-like resorption pattern on the alveolar bone surface, which is not usually detected in conventional radiographic imaging, but it may be noted during surgical excision of the cyst [3]. Although the gingival cyst is typically a solitary unicystic lesion, reports of multiples cysts have been described [4, 5]. It is most commonly found in patients in their fifth and sixth decades of life [3]. Histopathological features of this cyst consist in thin nonkeratinised epithelial lining with or without focal areas of acanthosis containing clear cells of glycogen-rich cytoplasm [4]. The present report describes an interesting case of a miniscule adult gingival cyst in the lower anterior gingiva associated with agenesis of multiple teeth to emphasize that such an association could go unnoticed due to the size of the lesion, thus raising the question as to how rare this odontogenic cyst really is.

## 2. Case Report

**Figure 1:**
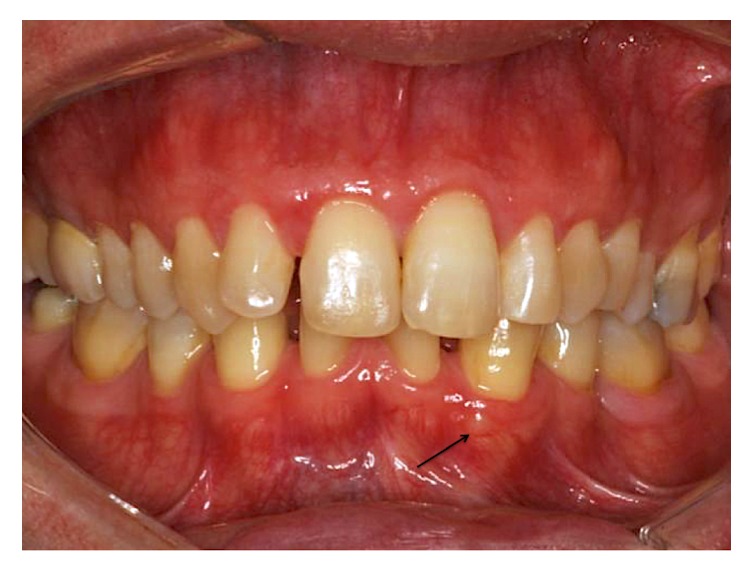
Clinical aspect of the lesion (arrow), showing the very small size of the lesion, denoting the difficulty in spotting it without thorough examination.

**Figure 2:**
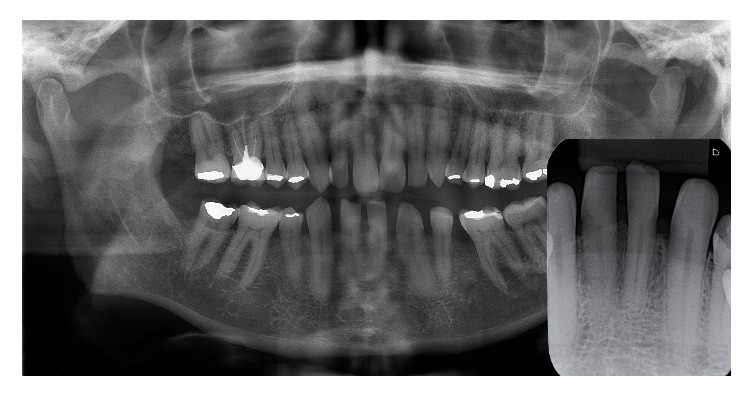
Panoramal radiograph showing agenesis of the lower lateral incisors and premolars. Inset periapical radiograph showing no evidence of bone involvement.

A 51-year-old male presented to a private orthodontic practice for initial assessment. His medical history was unremarkable and he was not on any medication at that time. During intraoral examination, a 1 × 1 mm translucent nodule with a smooth round surface was noted. It was located in the attached gingiva between the lower left mandibular canine and first incisor. The nodule was asymptomatic, cystic in appearance, fluctuant, and noncompressible (Figure 1). Pulp testing of his lower left canine and central incisor indicated normal pulp vitality. Radiographically, there was no finding suggestive of osseous involvement. The patient was unaware of the lesion prior to this assessment. Complementing his oral assessment, he also had bilateral agenesis of his lower lateral incisors and first premolars (Figure 2). Based on the clinical and radiographic findings, the provisional diagnoses were salivary gland tumor or cystic lesion of the gingiva.

**Figure 3:**
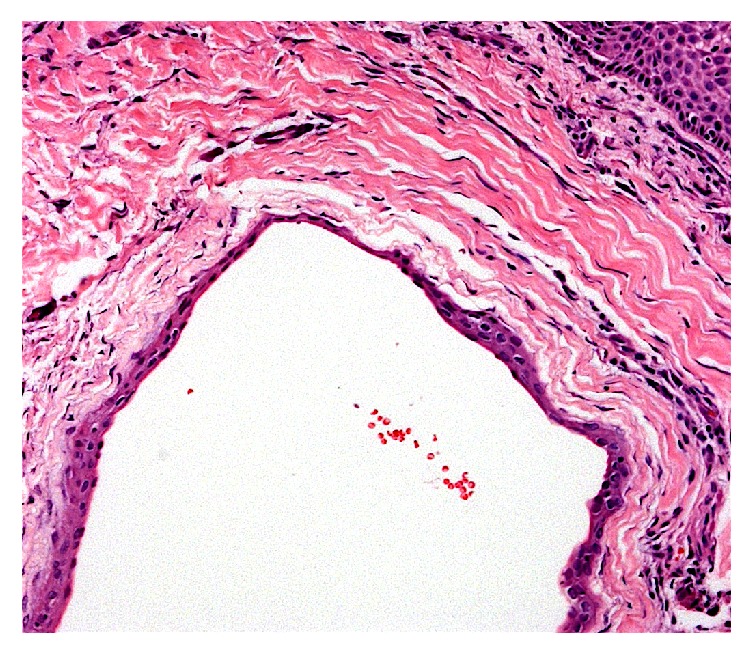
Cystic cavity lined by a thin and flattened epithelial lining.

**Figure 4:**
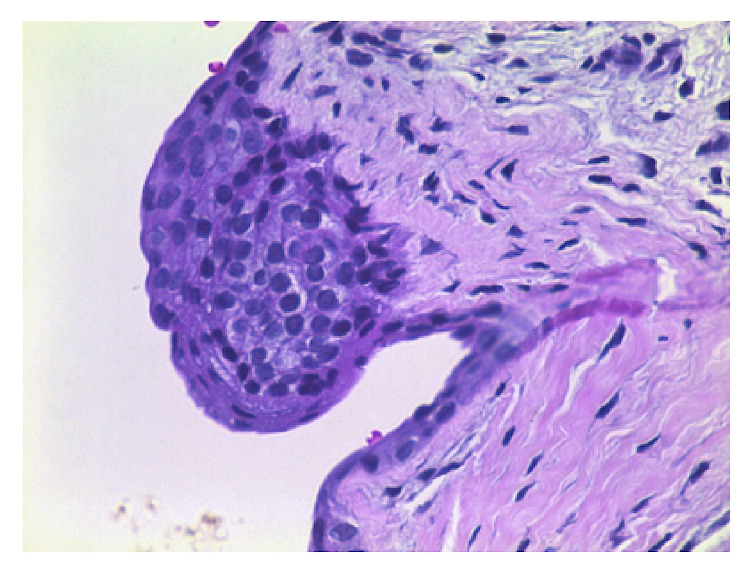
High-power showing in detail a plaque of the epithelial lining.

Due to the size of the lesion, an excisional biopsy was performed under local anesthetic infiltration. An incision was made in the overlying mucosa and the lesion was completely dissected from the adjacent tissues. The specimen was fixed in 10% buffered formalin and sent to the Oral Pathology Laboratory of the São Leopoldo Mandic Dental Institute and Research Center for histopathologic examination. On macroscopic inspection, the material consisted of a nodular fragment of soft tissue, measuring 5 × 3 × 2 mm, with smooth surface, whitish coloration, and friable consistency. Histological sections revealed a cystic cavity lined by two or three layers of odontogenic epithelium (Figure 3), exhibiting focal epithelial plates containing clear cells (Figure 4). The histopathologic diagnosis was of a gingival cyst of the adult.

The sutures were removed 1 week postoperatively and the patient reported minimal discomfort. After one month, the patient returned for follow-up when complete resolution of the lesion was observed. The patient is currently under regular follow-up with satisfactory healing and no relapse for over 15 months. Written consent was obtained from the patient for publication of the images relating to his case.

## 3. Discussion

The gingival cyst of the adult is an uncommon developmental odontogenic cyst that occurs on the gingiva or alveolar mucosa. Because of the clinical and morphologic similarities of this cyst and lateral periodontal cyst, Wysocki et al. in 1980 suggested that these two cysts shared a common histogenesis and the gingival cyst of the adult represents the extraosseous counterpart of the lateral periodontal cyst. The authors concluded that these two lesions arise from rests of the dental lamina [11]. Moskow and Bloom in 1983 reported essentially the same conclusions [1].

The gingival cyst of the adult and lateral periodontal cyst really share some clinical features, such as anatomic site of occurrence, age predilection, and histological features. Consequently, many reports in the past have combined these two entities interchangeably. Therefore, only very few studies documenting a real gingival cyst of the adult exist. Furthermore, virtually all gingival cysts of the adult are asymptomatic, which may well go unnoticed, especially if lesions are as small as the case presented herein, therefore unlikely to hit the pages of a publication. Wysocki et al. reported 10 cases with only soft tissue lesions in the gingiva with no bone involvement [11]. Cairo et al. reported 3 cases and emphasized the need for radiographic examination to differentiate gingival cysts of the adult from lateral periodontal cysts [12]. Giunta in 2002 reported a series of 22 gingival cysts of the adult in 21 patients observed over a period of 10 years and compared these cases with others from the literature providing more information about this cyst [5].

Gingival cysts of the adult occur much more frequently in the mandible than in the maxilla (Table 1), particularly in the premolar and canine regions [2, 3]. Seven cysts of the series by Wysocki et al. were in the premolar and canine areas of the mandible, one in the lateral incisor area of the maxilla, and the location of the remaining two is unknown [11].

The age distribution for gingival cysts of the adult is in fifth and sixth decades of life (Table 1). The review by Giunta of 94 cases, including Giunta's own report of 22 cases, showed that very few occurred in the first decade of life, whilst 77% occurred in the fourth, fifth, and sixth decades [5]. In the study by Jones et al., the patients diagnosed with gingival cyst of the adult were aged from 23 to 70, with mean of 52,9 ± 12,2 years [6]. In the present report, the patient was 51 years old and the cyst was located in the attached gingiva between the mandibular left canine and first incisor, as expected according to the literature reviewed herein.

Normally, patients with a gingival cyst of the adult have a history of a slow growing painless swelling [13]. The lesion is often less than 6 mm in diameter and may present the same color as the adjacent normal mucosa or even a bluish hue due to the cystic fluid [2]. In the case presented herein, the cyst was less than 2 mm in diameter and translucent pink in appearance. Furthermore, agenesis of the lateral incisors bilaterally coincided with the presence of the cyst. As this lesion is described to derive from the odontogenic epithelium, one cannot help but speculate whether in this case the lesion derived from the rests of the odontogenic epithelium that would have given rise to the missing lateral incisor. Sadly, none of the articles reviewed (Table 1) reported such an association, which is not the same as to say that it does not exist. The lesion in this case report was so inconspicuous that it could have easily gone unnoticed. This fact alone raises the questions as to how rare this lesion actually is, since such small cyst could be interpreted clinically as a variation of the normal mucosa and go unreported, thus masking its real prevalence.

Treatment of gingival cysts of the adult is primarily surgical excision, which carries a good prognosis, as no case has yet been reported of postoperative relapse. In the case presented herein, a follow-up of 15 months has been associated with no recurrence, as suggested by the literature reviewed.

In conclusion, this case report highlights the importance of a careful clinical examination to investigate subtle mucosal alterations, especially in areas of missing teeth, which may harbor potential lesions of odontogenic origin.

## Figures and Tables

**Table 1:** Published case reports and literature reviews on gingival cysts of the adult.

Authors (year)	*N*	Female	Male	Mandible	Maxilla	Mean age	Age range
Ramfjord (1953)	1	1	0	NR	NR	60	—
Ritchey and Orban (1953)	8	3	5	5^*∗*^	2^*∗*^	34	19–51
Bhaskar & Laskin (1955)	3	3	0	3	0	47	38–60
Kennedy (1957)	1	0	1	0	1	30	—
Holder & Kunkel (1958)	1	0	1	1	0	40	—
Bruce (1962)	2	2	0	2	0	52	31–60
Sherman (1963)	1	1	0	1	0	58	—
Grand & Marwah (1964)	1	NR	NR	0	1	62	—
Mabile (1965)	1	1	0	1	0	49	—
Alexander & Griffith (1966)	2	0	2	2	0	45	31–60
Zerden (1966)	2	0	2	2	0	52	—
Amar (1966)	1	1	0	1	0	19	—
Henning (1968)	1	1	0	1	0	53	—
Reeve & Levy (1968)	4	3	1	4	0	61	55–67
Young et al. (1972)	1	0	1	1	0	68	—
Laskaris & Skouteres (1975)	1	0	1	1	0	51	—
Moskow and Weinstein (1975)	3	*∗*	2	3	0	NR	—
Mesa (1976)	2	2	0	1	1	45	34–57
Bell et al. (1977)	8	7	1	7	1	51	35–65
Bucher and Hansen (1979)	33	19	14	24	9	48	7–77
Wysocki et al. (1980)	10	4	5	7	1	51	41–75
Brannon and Brasher (1981)	1	0	0	0	1	40	—
Gregg and O'Brien (1982)	2	1	1	1	1	52	35–68
Wescott et al. (1984)	2	0	2	2	0	46^*∗*^	—
Laskaris (1984)	3	2	1	2	1	54	42–63
Palattella et al. (1984)	1	0	1	1	0	29	—
Papanicolaou et al. (1986)	1	0	1	1	0	60	—
Shade et al. (1987)	2	0	2	2	0	47^*∗*^	—
Dent et al. (1990)	2	*∗*	1	2	1	64^*∗*^	—
Nxumalo & Shear (1992)	14	7	7	8	6	50	—
Haring, 1994	1	1	0	1	0	47	—
Fardal and Johannessen (1994)	1	1	0	1	0	41	—
Tolson et al. (1996)	1	0	1	1	0	50	—
Bell et al. (1997)	8	7	1	7	1	51	35–65
Cairo et al. (2002)	3	3	0	3	0	46	27–59
Giunta (2002)	22	15	6	16	2	52	32–80
McGuff et al. (2003)	1	0	1	1	0	45	—
Hegde & Reddy (2004)	1	0	1	1	0	18	—
Cunha et al. (2005)	1	1	0	1	0	69	—
Damm and Fantasia, 2006	1	0	1	0	1	44	—
*Jones et al. (2006) [6]*	16	12	4	NR	NR	52.9	SD = 12.2
*Sato et al. (2007) [2]*	1	0	1	1	0	78	—
Noonan et al. (2008)	1	NR	NR	1	NR	NR	—
Kelsey et al. (2009)	1	0	1	1	0	54	—
*Malali et al. (2012) [7]*	1	0	1	0	1	16	—
*Agostini et al. (2015) [8]*	1	1	0	0	1	35	—
*Gil Escalante and Tatakis (2015) [9]*	1	0	1	1	0	46	—

Table adapted from Giunta 2002 [5] and Kelsey et al. 2009 [10] and references therein. References not cited in Giunta 2002 and Kelsey et al. 2009 are shown in italics.

__^*∗*^Information missing on 1 or more cases. NR = not reported. SD = standard deviation.
